# A case of phentermine-induced psychosis: the need for caution for drug-drug interactions

**DOI:** 10.1192/j.eurpsy.2023.2157

**Published:** 2023-07-19

**Authors:** S. Sreevalsam Anil, M. Thootkur, J. White

**Affiliations:** 1 Psychiatry, Carilion Clinic-Virginia Tech Carilion School of Medicine, Roanoke, United States

## Abstract

**Introduction:**

Phentermine is a sympathomimetic amine that the U.S Food and Drug Administration has approved for short-term use in the treatment of obesity. However, there have been case reports of phentermine being associated with neuropsychiatric symptoms, and thus caution is needed to avoid drug-drug interactions when prescribing phentermine (Nathan PJ, *et al*. CNS Neurosci Ther 2011;17:490-505) We present a case of phentermine-induced psychosis that could have been precipitated after being co-prescribed with fluoxetine.

**Objectives:**

To discuss a case of phentermine-induced psychosis that could have been precipitated by CYP3A4 inhibition of phentermine by fluoxetine.

**Methods:**

Miss X is a 61-year-old female with a history of major depressive disorder, generalized anxiety disorder, obesity, and rheumatoid arthritis. Her psychiatric symptoms were stable with oral fluoxetine 60 mg daily, oral aripiprazole 2mg daily, oral amitriptyline 100mg at night, and oral lorazepam 1mg daily. Miss X was prescribed oral phentermine 15mg daily for appetite suppression for weight loss. Subsequently, she started developing paranoid delusions against her family members, generalized anxiety, increased psychomotor activity, decreased appetite, and decreased sleep. Her symptoms continued to worsen even after discontinuing her medications on the 7th day. Miss X was eventually brought to the emergency room on the 14th day as her symptoms continued to deteriorate and she could not take care of herself.

**Results:**

Miss X’s symptoms resolved after a dose of Intramuscular injection of 2mg of lorazepam. No signs of serotonin syndrome were present during the examination. Drug-drug interaction between phentermine and fluoxetine is suspected to be a causative factor in the precipitation of psychosis as fluoxetine can inhibit the CYP3A4 metabolism of phentermine. Her electrocardiogram also demonstrated prolonged QTc (470ms), which could have been precipitated by co-prescribing phentermine and amitriptyline. Miss X was admitted to the inpatient psychiatric unit, and oral fluoxetine 60mg daily, oral aripiprazole 2mg daily, and oral lorazepam 1mg daily were restarted. Due to QTc prolongation oral trazodone 50mg daily was started instead of amitriptyline. After her psychiatric symptoms were stable on the medication regimen, Miss X was discharged on the third day of admission to the inpatient psychiatric unit.

**Conclusions:**

Our case demonstrates the caution needs to be taken when prescribing phentermine not only for its neuropsychiatric side-effects but also for drug-drug interactions.

**Disclosure of Interest:**

None Declared

